# HIV-Related High-Risk Behaviors among Chinese Migrant Construction Laborers in Nantong, Jiangsu

**DOI:** 10.1371/journal.pone.0031986

**Published:** 2012-03-30

**Authors:** Xun Zhuang, Zunyou Wu, Katharine Poundstone, Changqing Yang, Yaqin Zhong, Shengyang Jiang

**Affiliations:** 1 National Center for AIDS/STD Control and Prevention, Chinese Center for Disease Control and Prevention, Beijing, China; 2 Department of Epidemiology and Biostatistics, School of Public Health, Nantong University, Nantong, Jiangsu Province, China; Vanderbilt University, United States of America

## Abstract

**Background:**

HIV transmission in rural areas of China is being fueled in part by migrant workers who acquire HIV outside of their hometowns. Recent surveillance statistics indicate that HIV prevalence among returning migrants has increased significantly.

**Methods:**

We conducted a community-based cross-sectional study to assess HIV-related knowledge, attitudes and behaviors among migrant returnees in Nantong, Jiangsu Province, one of the largest exporters of migrant laborers.

**Results:**

A total of 1625 subjects were enrolled with a response rate of 89%. All participants were male and of the majority Han ethnicity. The mean age was 39.0 years (SD = 6.7; range: 18 to 63), and most had a stable partner (N = 1533, 94.3%). Most correctly identified the major modes of HIV transmission (68.9%–82.0%), but fewer were able to identify ways that HIV cannot be transmitted. Nearly one-third of participants held positive attitudes toward having multiple sex partners, and nearly half believed that sex work should be legalized. Multiple logistic regression analysis indicated that risky sexual behavior (defined as sex with a casual or commercial sex partner) was associated with no stable partner; working abroad; correct condom use; age <22 at first sex; higher coital frequency; and having a positive attitude towards multiple sex partners.

**Conclusions:**

We found high levels of reported sex with a casual or commercial sex partner and low levels of consistent condom use. HIV prevention interventions among migrant workers need to focus on younger migrants, migrants without stable partners, and migrants who travel abroad for work.

## Introduction

China's rapid economic development has been fueled by an unparalleled migration of rural residents to urban centers. There were an estimated 140 million migrant laborers in China by the end of 2008 [1]. Nearly two thirds of China's migrants are male, and the vast majority (86%) are under the age of 40 years old [2], [3].

Of the many public health consequences of migration, one major concern is HIV vulnerability among male migrant laborers. ‘Oscillating’ or ‘circular’ migration, in which migrants leave home and return over regular intervals, has been associated with increased HIV prevalence among male migrants in South Africa [4], [5], Senegal [6] and Nepal [7]. Limited education, constant mobility, hazardous working conditions, low wages, chronic underemployment, and substandard housing are also factors that can compromise the health of migrant laborers and make them more vulnerable to HIV [4], [8], [9].

Oscillating migration is also the most common form of migration in China, where many migrants return home for a month around the Chinese New Year holiday. Separated from their families and social support for the rest of the year, many migrants engage in sexual behaviors that put them at risk for HIV [10], [11], [12], [13], [14]. Male migrants are also more likely than non-migrants to have someone in their social network that has multiple sex partners or has sex with sex workers [15], [16], [17].

Most research in China on HIV among migrant workers has been conducted in large cities that migrants travel to in search of work [10], [13], [18], [19], [20]. We conducted a community-based cross-sectional study of HIV-related risk behaviors among rural migrants in Nantong, Jiangsu Province, one of the largest exporters of migrant laborers. Every year, a large proportion of Nantong's rural population migrates to other cities to do non-agricultural work, and recent reports indicated that HIV prevalence among returning migrants has increased significantly [21]. The aim of our study was to identify factors associated with sexual risk taking in this population.

## Methods

### Study site

Nantong has a population of 7.8 million, and it exports the largest number of laborers in Jiangsu Province. Every year, around 550,000 Nantong residents leave home in search of work, and another 15,000 leave the country to work abroad. The vast majority of laborers leaving Nantong (80%) are engaged in construction work.

The first HIV case in Nantong was reported in 1998. By December 2009, 332 HIV cases had been reported. Of these, 84 were migrant returnees to Nantong, and 25 were women infected by their migrant returnee husbands. Most of the HIV-positive migrant returnees were construction workers, some of whom had worked abroad.

### Study Population

Between January and June 2007, we recruited male migrant laborers from Xingfu Township, Nantong Prefecture, and several services companies that hire male migrants from Nantong. Individuals were eligible if they were male, and if they were migrant laborers, which we defined as being registered as a permanent resident of Nantong but involved in work outside of Nantong.

Of the 1825 migrants who volunteered to participate in the study, 1625 (89.0%) were eligible for participation. Of the 1625 eligible participants, 466 (28.7%) had returned to Nantong after working abroad within the past six months. Each was paid the equivalent of US$ 1.50 for study participation.

### Ethics

The study was reviewed and approved by the Institutional Review Board (IRB) of National Center for AIDS/STD Control and Prevention, the Chinese Center for Disease Control and Prevention. The objectives and the procedure of the study, and potential risks and benefits of participating in the study were given to potential participants during the recruitment of study subjects. Written informed consents were obtained from all subjects participated in the survey.

### Data collection

After being briefed about the research study, participants were asked to complete an anonymous questionnaire covering socio-demographic characteristics and HIV/AIDS knowledge, attitudes and behaviors. Those who were illiterate (n = 12) opted for a face-to-face interview with trained interviewers from the Nantong University School of Public Health.

#### Sociodemographic Characteristics

Demographic information collected included: age; gender; ethnicity (Han or Other); marital status (currently married, remarried, cohabitation, single, divorced and widowed); occupation (general worker and manager); educational level (illiterate/elementary school, junior high school, senior high school); and monthly income (RMB <1500, 1500–2000, >2000). Marital status was collapsed into two categories: stable partner and no stable partner. Participants were also asked about their living and working conditions, which included working place, daily working hours, vacation days per month, and family income.

#### Knowledge

HIV/AIDS knowledge was assessed through 8 items, including:

1. Can a person be infected with HIV if you inject blood or blood products contaminated by HIV virus?
2. Can a person be infected with HIV if you share needles with HIV-infection?
3. Can a baby be born with HIV if the baby's mother is HIV-positive?
4. Can a person reduce their risk of HIV infection if he/she remains faithful to one sexual partner?
5. Can a person reduce their risk of HIV infection if they use a condom during sex?
6. Can a person be infected with HIV if they share a meal with someone who is HIV-positive?
7. Can someone with HIV infection look like a healthy person?
8. Can a person be infected with HIV if they are bitten by mosquito?

These items were adapted from the scales used in China's National HIV Surveillance Surveys, and are consistent with measures used in other studies in China [22], [23].

#### Attitudes

Participants were asked whether they agreed or disagreed with the following statements: 1) “It is acceptable for people to have multiple sexual partners,” and 2) “Commercial sex should be legalized.” Participants were also asked whether they perceived themselves to be at risk of HIV infection (“possible” or “impossible”).

#### Behaviors

Participants were asked about their sexual activity; condom use with stable/casual/commercial partners (never, sometimes, always); drug use (never, past drug use or current drug use). Participants were also asked whether or not they had sold their blood or plasma. Participants who had sexual experience were also asked about age at marriage; age at first sex; sexually transmitted disease history; site of casual/commercial sex activities; and frequency of sexual activities with their stable partner.

### Statistical analysis

We conducted descriptive analyses to describe the characteristics of our study population. We created a summary score for HIV/AIDS knowledge, with 2 points assigned for each correct answer, 1 point for each answer stating “not sure” or “unknown” and 0 points for each incorrect answer, giving a range from 0 to 16. Tests of associations between certain categorical variables were based on the chi-square test. Univariate logistic regression analyses were performed to provide unadjusted crude parameter estimates. Variables significant at α = 0.10 in the univariate analyses were included in our multivariable logistic regression analysis. We performed a multivariable logistic regression (backward method) to measure associations between risk factors and ever having had sex with casual partner or commercial sex partner. Odds ratios (ORs) and 95% confidence intervals were calculated. All statistical analyses were carried out using the SPSS 13.0 for Windows (SPSS Inc., Chicago, IL).

## Results

### Characteristic of participants

All participants were male and of the majority Han ethnicity (**Table 1**). The mean age of the participants was 39.0 years (SD = 6.7; range: 18 to 63), and most had a stable partner (N = 1533, 94.3%). The majority of participants were general construction workers (N = 1454, 89.5%), and only 17.5% had completed senior high school or above. Median monthly income was 2100 Chinese Yuan (Range: 850 to 30,000). Most had worked as migrant laborers within China (N = 1159, 71.3%), while 466 (28.7%) had worked in foreign countries. Among those who had worked in foreign countries, 40.4% had worked in Russia, 16.5% in Singapore, 8.3% in Algeria, 3.7% in United Arab Emirates, and 31.1% in other countries.

**Table 1:** Socioeconomic and demographic characteristics of study participants.

Characteristic variables	N	%
Age (Years)		
<30	187	11.5
30–39	768	47.3
40–49	604	37.2
≥50	66	4.1
Current marital status		
Stable partner	1533	94.3
No stable partner	92	5.7
Occupation		
Worker	1454	89.5
Manager	78	4.8
Other	93	5.7
Education		
Elementary/Illiterate	232	14.2
Junior high school	1109	68.2
Senior high school	284	17.5
Monthly income (CNY*)		
<1500	115	7.1
1500–2000	672	41.3
>2000	838	51.6
Working place		
Domestic	1159	71.3
Foreign	466	28.7
Family economic situation		
Low	481	29.6
Middle	1023	63.0
High	121	7.4

* CNY: Chinese Yuan; 6.5 CNY ≅ 1 USD.

### Knowledge

Most participants correctly identified the major modes of HIV transmission (68.9%–82.0%), but fewer were able to identify ways that HIV cannot be transmitted (**Table 2**). Nearly one-half (44.2%) thought that HIV can be contracted from eating, and about one third (36.3%) reported that a person living with HIV can appear healthy. Only 13.3% thought that HIV could not be contracted from eating with an HIV positive person.

**Table 2:** HIV/AIDS-related knowledge among study participants.

Item	Number of correct responses (N = 1625)
1) Blood/blood product transmission	1333 (82.0%)
2) Needle sharing	1303 (80.2%)
3) Mother-to-child transmission	1285 (79.1%)
4) Sexual transmission	1160 (71.4%)
5) Condom protection	1119 (68.9%)
6) Eating together	718 (44.2%)
7) HIV-positive individuals can appear healthy	590 (36.3%)
8) Mosquito bite	216 (13.3%)

### Attitudes

Most participants did not agree that with the statement “It is acceptable for people to have multiple sexual partners” (N = 1114, 68.6%), while 787 (48.4%) of participants agreed with the statement “Commercial sex should be legalized.” Over two-thirds of study participants perceived themselves to be at risk of HIV infection (N = 1154, 71.0%).

### Sexual behavior

Nearly all participants reported being sexually active (N = 1546, 95.1%), and most were married (88.0%). The mean age at first sexual intercourse was 23.9 years, and the mean age at marriage was 24.5 years. Premarital sex was more prevalent in the youngest age group compared with the oldest age group (75% among those under 30 versus 15% among those 50 and older). Among married participants, 75.0% reported having had only one sexual partner in their lifetime, and 98% reported sexual intercourse with their wife during the past year. However, 15.8% of married participants reported having had casual extramarital sex during the past year, and most of those having casual extramarital sex were between 30–40 years old (54.3%). Casual sex was also more prevalent among participants who had returned from abroad compared with those who had worked domestically (18.2% versus14.9%), though the difference was not statistically significant (χ^2^ = 3.111, *p* = 0.211).

About 14.2% of participants reported having had sex with a sex worker. Among those who had returned from abroad, the rate was slightly higher compared with those who had worked domestically (17.4% versus 12.9%, χ^2^ = 6.764, *p* = 0.034). The most commonly cited reasons for sex with a sex worker were: meeting physical needs (56.8%), loneliness (15.4%), looking for excitement (15.4%), curiosity (13.7%), and peer influence (10.6%).

Participants were also asked how frequently they had sex when they were at home (coital frequency). Younger participants reported more frequent sexual intercourse than older participants. Most participants (58.6%) reported 1–2 times per week, 22.7% reported less than 1 time per week, 16.5% reported 3–4 times per week and 4.0% reported more than 4 times per week.

Almost 90.8% reported that they heard of condoms, and 59.3% said that they knew how to use a condom correctly. Most participants with stable partners had never used condoms during sex with their wives or stable partners. Among those having sex with sex workers, 21.3% had never used a condom; 43.3% sometimes used a condom; and 35.4% reported consistent condom use. Among those reporting sex with a casual partner, 13.8% had never used condoms; 37.8% sometimes used a condom, and 48.4% reported consistent condom use. The main reason cited for condom use with a stable partner was contraception, while the main reason cited for condom use with a sex worker or casual partner was to protect against disease.

### Risk factors for ever having had sex with a casual or commercial sex partner

In univariate analyses, our primary outcome, ever having had sex with a casual or commercial sex partner, was significantly associated with young age; no stable partner; reporting manager as occupation; working abroad; age <22 at first sex; higher coital frequency; correct condom use; and having a positive attitude towards multiple sex partners (**Table 3**). All factors significant at α = 0.10 in univariate analysis were included in the multiple regression analysis. After controlling for potential confounding variables, results from multiple logistic regression analysis indicated that ever having had sex with a casual or commercial sex partner was associated with having no stable partner; working abroad; self-report of knowing how to correctly use a condom; being under the age of 22 at first sex; having a higher coital frequency; and having a positive attitude towards multiple sex partners (**Table 4**).

**Table 3:** Univariate analyses of factors associated with ever having had sex with a casual or commercial sex partner (“Higher risk sex”).

Variable	N	Higher risk sex		p-value	OR (95%CI)
		Yes	%		
Age (Years)					
<30	154	44	28.6	0.001	1
30–39	740	184	24.9		0.83 (0.56, 1.22)
40–49	588	114	19.4		0.60 (0.40, 0.90)
50+	64	9	14.1		0.41 (0.19, 0.90)
Current marital status					
No stable partner	70	25	35.7	0.044	1
Stable partner	1476	326	22.1		0.62 (0.39, 0.99)
Education					
Elementary/Illiterate	230	48	20.9	0.751	1
Junior high school	1083	248	22.9		1.13 (0.80, 1.60)
Senior high school	233	55	23.6		1.17 (0.76, 1.82)
Monthly income (CNY)*					
<1500	111	19	17.1	0.384	1
1500–1999	659	153	23.2		1.46 (0.87, 2.48)
2000+	776	179	23.1		1.45 (0.86, 2.45)
Occupation					
Worker	1454	321	22.1	0.075	1
Manager	78	26	33.3		1.76 (1.08, 2.87)
Other	93	21	22.6		1.03 (0.62, 1.70)
Working place					
Foreign	416	107	25.7	0.086	1
Domestic	1130	244	21.6		0.80 (0.61, 1.03)
HIV knowledge score					
≥12	854	213	24.9	0.517	1
<12	692	159	23.0		0.91 (0.51, 1.52)
Commercial sex should be legalized.					
Agree	738	181	24.5	0.102	1
Disagree	808	170	21.0		0.82 (0.65, 1.04)
Perceived HIV risk					
Possible	1097	259	23.6	0.184	1
Impossible	449	92	20.5		1.20 (0.92, 1.57)
Age at first sex**					
<22	245	99	40.4	<0.001	1
≥22	1284	250	19.5		0.36 (0.27, 0.48)
Coital frequency (per week)					
<1	347	78	22.5	0.007	1
1–2	868	177	20.4		0.88 (0.65, 1.19)
3–4	252	75	29.8		1.46 (1.01, 2.11)
>4	61	19	31.1		1.56 (0.86, 2.84)
Use condom correctly					
Yes	894	252	28.2	<0.001	1
No	652	99	15.2		0.46 (0.35, 0.59)
STD*** history					
Yes	57	15	26.3	0.508	1
No	1489	336	22.6		0.82 (0.45, 1.49)

* CNY: Chinese Yuan; 6.5 CNY≅1 USD.

** In China, 22 years is youngest legal age for marriage.

*** STD: Sexually Transmitted Disease.

**Table 4:** Multivariable logistic regression analysis of factors associated with ever having had sex with a casual or commercial sex partner.

Variable	p-value	OR 95% CI
Marital status		
No stable partner	0.004	1
Stable partner		0.34 (0.16, 0.71)
Working place		
Foreign	0.043	1
Domestic		0.75 (0.56, 0.99)
Self-report of correct condom use		
Yes	<0.001	1
No		0.47 (0.36, 0.63)
Age at first sex		
<22	<0.001	1
≥22		0.44 (0.32, 0.60)
Coital frequency (times/week)		
<1	0.043	1
1–2		0.80 (0.58, 1.10)
3–4		1.25 (0.84, 1.87)
>4		1.30 (0.69, 2.45)
Attitude towards multiple sex partners		
Agree	0.002	1
Disagree		0.65 (0.50, 0.85)

## Discussion

Male, rural laborers who migrate to urban areas and foreign countries in search of work have the potential to bring HIV with them when they return home. Removed from their usual social networks and far from home, many male migrant laborers have sex with casual or commercial sex partners, placing them at increased risk for HIV. In China, surveillance statistics indicate that HIV rates are increasing in many rural areas in which a significant proportion of the population migrates for work.

In our sexually active study population consisting of male migrant laborers from Nantong, Jiangsu Province, we found high levels of reported sex with a casual or commercial sex partner; low levels of consistent condom use with casual or commercial sex partners; and extremely low levels of condom use among those with stable partners. Local statistics indicate that migrant workers and their stable partners account for nearly one-third of reported HIV cases in Nantong, making migrant workers an important target population for HIV prevention efforts.

Our study findings have important implications for the development of HIV prevention interventions among migrant workers in Nantong. First, we found that migrants without a stable partner were more likely to have reported higher risk sex, suggesting the importance of focusing HIV prevention interventions on migrants without a stable partner. Second, we found that migrants traveling abroad for work were more likely to report higher risk sex. Migrants traveling to higher HIV and STD prevalence areas are at increased risk of HIV exposure should they engage in high risk behaviors. Migrants traveling abroad need to know how to avoid acquiring or transmitting HIV infection. Third, we found that younger age at first sex was associated with higher risk sex and a higher number of lifetime sex partners. Similar findings have been reported in other studies [24], [25], [26]. Promotion of a later sexual debut can help support HIV prevention efforts and can be included as a component of future HIV prevention interventions in this population. Fourth, participants who agreed with the statement “It is acceptable for people to have multiple sexual partners” were more likely to report higher risk sex. HIV prevention interventions should underscore the importance of partner reduction and consistent condom use with casual or commercial sex partners. Fifth, many gaps in HIV knowledge remain, and future HIV education programs for migrants should attempt to eliminate misconceptions about HIV transmission and increase knowledge of condoms and proper condom use. Finally, HIV education and prevention interventions among migrant workers must take into account lower levels of literacy.

Our study has several limitations. First, this was a cross-sectional survey, limiting our ability to draw causal inferences. Second, because sex remains a sensitive topic in China, our findings may be biased by under- or over-reporting of sexual risk behaviors. Third, our findings may be subject to recall bias. To mitigate these risks, we carefully selected and trained interviewers; we used a self-administered questionnaire (with face-to-face interviews limited to those who were illiterate); and provided assurances of anonymity.

Most previous studies in China have focused on urban areas that are the destinations for migrant workers. Given the oscillating nature of migration in China, these studies provide only a partial picture of HIV risk among migrant worker populations. Our study and recent HIV surveillance statistics from Nantong underscore the importance of including rural areas that are the source of migrant workers in HIV prevention and treatment programs. HIV prevention programs for migrant workers should focus on areas from which migrants leave, as well as the workplaces to which migrants travel.
